# A94 EXPLORING THE SOCIOECONOMIC BURDEN OF PEDIATRIC INFLAMMATORY BOWEL DISEASE: A SURVEY OF FAMILIES AND PEDIATRIC PROVIDERS

**DOI:** 10.1093/jcag/gwac036.094

**Published:** 2023-03-07

**Authors:** T Desai, M Yaskina, M Carroll

**Affiliations:** 1 Pediatrics, University of Alberta; 2 Division of Pediatric Gastroenterology and Nutrition, Stollery Children's Hospital; 3 University of Alberta; 4 Women and Children’s Health Research Institute, Edmonton, Canada

## Abstract

**Background:**

Rates of pediatric inflammatory bowel disease (IBD) have been increasing significantly over recent decades, contributing to the rising chronic disease burden across pediatrics. Currently, there is very limited literature exploring the financial burden on families of children with IBD. In fact, there is only one Canadian study in this area despite Canada having one of the highest rates of pediatric IBD in the world.

**Purpose:**

The goal of this study is to better understand the socioeconomic burden of pediatric IBD at our institution and compare institutional practices across the country.

**Method:**

The study took place at a large, tertiary care pediatric centre in Edmonton, Alberta, Canada between October 2022 and January 2023. Two separate electronic surveys were developed and distributed to all families of children with IBD at our institution (N= ~400) and pediatric IBD providers across the country (N= ~45) respectively. Surveys explored demographic information, financial impacts of IBD diagnosis and perceptions around pediatric IBD care and financial support.

**Result(s):**

Interim results (N=3) indicate dietary therapy costs, missed time off work and school and time off for IBD treatment as considerable burdens on families. Across the country, initial provider data (N=6) suggests significant variability in clinical practice, allied health support and financial support for families. There is overwhelming agreement among providers that the socioeconomic burdens on families is significant. Further data and regression analysis is ongoing.

**Conclusion(s):**

This is the first study in Canada to directly explore the socioeconomic burden on families of children with IBD. Results indicate good correlation between provider awareness and the increased financial burden on families but also considerable variation in practice across the country. Data suggest further research and advocacy is required to better support patients, however various quality improvement opportunities exist both locally and beyond.

**Please acknowledge all funding agencies by checking the applicable boxes below:**

None

**Disclosure of Interest:**

None Declared

